# The London and Counties Medical Protection Society

**Published:** 1893-10-07

**Authors:** 


### The London and Counties Medical Protection Society.

At the last County of London Sessions, Abraham David Blumberg, described as a medical student of the London Hospital, pleaded guilty to having by false pretences obtained the sum of £2 from Jessie Donenberg with intent to defraud. Mr. Bodkin and Mr. Mallinson prosecuted for the London and Counties Medical Protection Society. Mr. Ernest Beard defended the prisoner. Mr. Bodkin said that although admitting that the prisoner had a certificate as a doctor in Russia he was still not entitled to represent himself as he did as a registered medical practitioner. He had a plate on his door, on which were the words, "Dr. Blumberg, M.D." Among his patients was Jessie Donenberg, on whom he had operated for the cure of sterility, with the result that her health was permanently damaged. In consideration, however, of the prisoner's pleading guilty and promising never again to practise as a doctor, also bearing in mind that he had already been imprisoned for about a month, the Society did not desire to press the charge against him, but would ask the Court to deal leniently with him. Mr. Beard gave an undertaking that his client would no longer practise as a doctor. His Lordship warned the prisoner that he had been guilty of a very grave breach of the law, but that on his undertaking not to offend again he (the Judge) would dea. with him under the First Offenders Act, and bind him over in the sum of £50 to come up for judgment if called upons His Lordship hoped that this prosecution would warn other- that they would render themselves liable to imprisonment if they obtained money by representing themselves to be registered medical practitioners.

